# Pseudo label refining for semi-supervised temporal action localization

**DOI:** 10.1371/journal.pone.0318418

**Published:** 2025-02-05

**Authors:** Lingwen Meng, Guobang Ban, Guanghui Xi, Siqi Guo

**Affiliations:** Electric Power Research Institute of Guizhou Power Grid Co. Ltd, Guiyang, China; University of Economics Ho Chi Minh City, VIET NAM

## Abstract

The training of temporal action localization models relies heavily on a large amount of manually annotated data. Video annotation is more tedious and time-consuming compared with image annotation. Therefore, the semi-supervised method that combines labeled and unlabeled data for joint training has attracted increasing attention from academics and industry. This study proposes a method called pseudo-label refining (PLR) based on the teacher-student framework, which consists of three key components. First, we propose pseudo-label self-refinement which features in a temporal region interesting pooling to improve the boundary accuracy of TAL pseudo label. Second, we design a module named boundary synthesis to further refined temporal interval in pseudo label with multiple inference. Finally, an adaptive weight learning strategy is tailored for progressively learning pseudo labels with different qualities. The method proposed in this study uses ActionFormer and BMN as the detector and achieves significant improvement on the THUMOS14 and ActivityNet v1.3 datasets. The experimental results show that the proposed method significantly improve the localization accuracy compared to other advanced SSTAL methods at a label rate of 10% to 60%. Further ablation experiments show the effectiveness of each module, proving that the PLR method can improve the accuracy of pseudo-labels obtained by teacher model reasoning.

## Introduction

Temporal Action Localization (TAL) is an important task in the field of video understanding. The goal of this task is to identify and locate the categories of all actions that appear in a video, as well as the corresponding start and end times of the actions. TAL technology has wide application value in video editing, sports game analysis, surveillance video processing, and other fields. In recent years, TAL technology has developed rapidly, and a number of high-performance localization models such as MTSN [1], Tallformer [2], Contextloc++ [3], ActionFormer [4] have emerged.

However, the training of these models relies on a large amount of manually annotated data. Labeling videos is complex, time-consuming, and expensive [5]. Therefore, in practical application scenarios, fully labeling all video data is not economically feasible. This has prompted the academic and industry communities to pay more attention to the application of semi-supervised learning (SSL) methods in the video field. Semi-supervised learning is a widely used training method in machine learning and deep learning, which features both labeled and unlabeled data in the training data. The purpose of semi-supervised learning is to fully explore the information of unlabeled data based on the training of labeled data.

Most existing semi-supervised learning methods follow the paradigm of self-training and consistency regularization [6–8]. Specifically, self-training methods are achieved by generating pseudo labels and using them to retrain the model, while consistency regularization is achieved by applying different levels of data augmentation and requiring consistent predictions. Data augmentation methods include masking, scaling, adding Gaussian noise, etc. Work in the field of semi-supervised temporal action localization [9, 10] also basically follows this paradigm.

**Fig 1:**
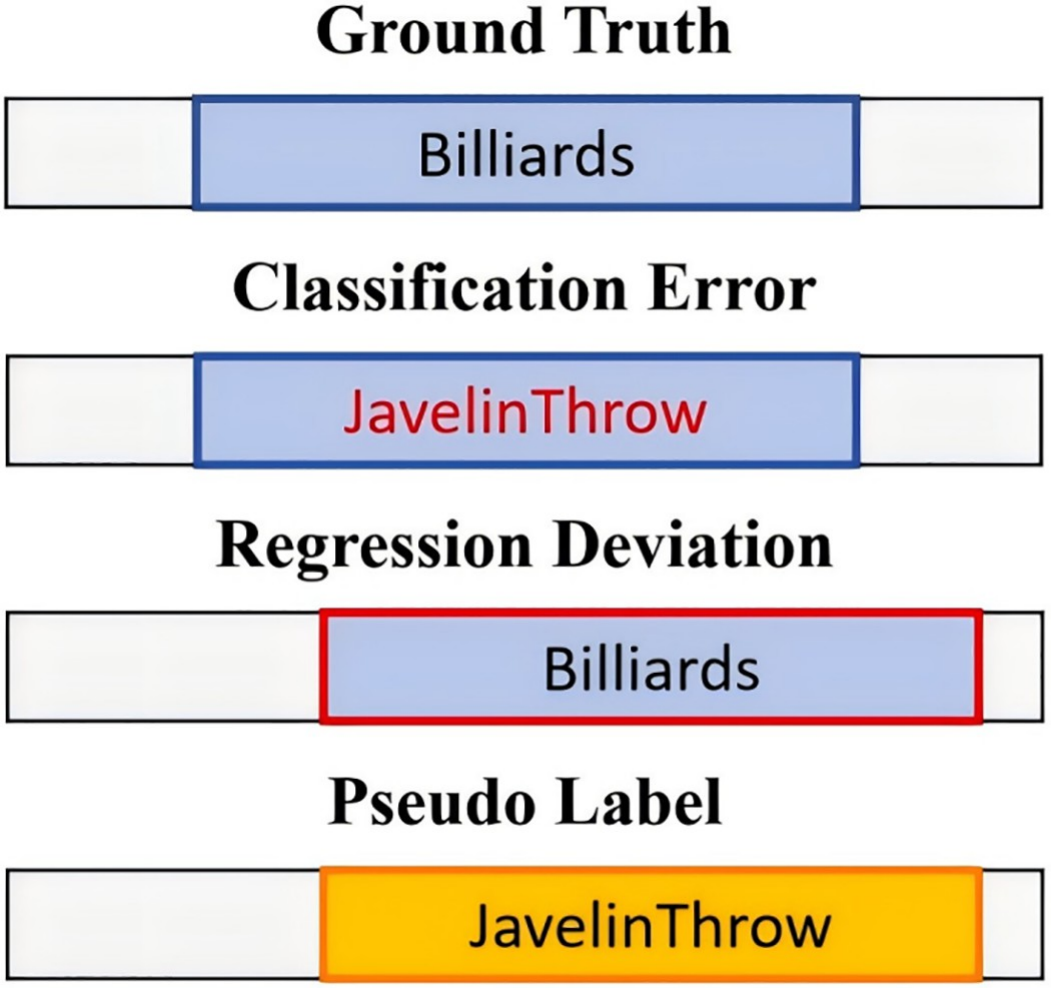
The illustration of noise in pseudo label. Pseudo label noisy consists of two parts, one is the classification error, the other is boundary offset.

However, these methods mainly focus on how to generate more accurate pseudo labels through more reliable feature representations, without paying attention to how to improve the quality of pseudo labels. The noise within pseudo-labels directly affects the effectiveness of semi-supervised learning, as shown in Fig 1. There are some attempts to alleviate the impact of false label noise through other means. NPL [5] attempts to aggregate classification and regression information to improve regression accuracy. SPOT [10] proposes a single-stage model which conducts classification and location simultaneously to generate pseudo labels. Their experimental results prove that integrating information from classification and regression branches can effectively improve the performance of semi-supervised learning.

However, they still did not directly optimize the generated pseudo labels. Therefore, we propose a framework to improve the temporal localization accuracy of pseudo-labels, called Pseudo-Label Refining (PLR), which consists of three parts: Pseudo-Label Self-Refinement (PLSR), Boundary Synthesis (BS) and Adaptive Weight Learning (AWL).

Firstly, PLSR is a module designed specifically for temporal action localization tasks. It refines initial pseudo labels through multiple rounds of self-tuning and boundary synthesis. By extracting features from unlabeled video segments, applying linear interpolation and 1D convolutional layers, PLSR produces a deeper representation. This representation is then fed into a classifier and regressor to obtain refined action boundaries and classification confidence scores. PLSR effectively adapts the concept of bounding box refinement from 2-stage object detection to SS-TAL, enabling fine-tuning of action intervals.

Secondly, BS aims to mitigate the negative effects of inaccurate pseudo labels by leveraging information from surrounding non-maximum predictions. It achieves this through three steps: randomly disturbing existing action intervals, adding Gaussian noise to the corresponding features, and averaging the results of multiple predictions weighted by their confidence scores. This mechanism ensures that the final refined action boundaries incorporate valuable information from multiple perspectives, improving the robustness and accuracy of temporal action localization.

Finally, AWL tackles the issue of varying learning difficulties among unlabeled samples and the unreliability of pseudo labels generated by the teacher model. It assigns dynamic weights to pseudo labels based on their credibility, as measured by the averaged information entropy. During training, the model prioritizes learning from high-confidence labels while gradually incorporating information from lower-confidence ones. This adaptive approach enhances the overall performance and robustness of the temporal action localization model by allowing it to focus on more reliable data while still benefiting from the full range of unlabeled data.

The proposed method is tested on the THUMOS14 [11] and ActivityNet v1.3 [12] datasets, and the experimental results show that the method trained with PLR outperforms the previous state-of-the-art methods by up to 3.5% mAP under four different label rates: 10%, 20%, 40%, and 60%.

Our contributions can be divided into three points:

- We propose the Pseudo Label Self Refinement and Boundary Synthesis module tailored for temporal action localization. PLSR adapts bounding box refinement to the temporal domain for fine-tuning action intervals, as well as BS mitigates inaccuracies by leverages multiple inference, improving pseudo label accuracy.
- We propose Adaptive Weight Learning for Performance Boost. AWL dynamically weights pseudo labels based on credibility, prioritizing high-confidence labels and gradually incorporating lower-confidence ones.
- Extensive experiments conducted on two major benchmarks, THUMOS14 [11] and ActivityNet v1.3 [13], demonstrate that PLR effectively utilizes unlabeled data to enhance temporal action localization performance, outperforming prior state-of-the-art methods.

## Related work

### Temporal action localization

Temporal Action Localization (TAL) methods [14] are typically categorized into three types: two-stage, single-stage, and anchor-free methods. Two-stage methods [13, 15–19] initially generate candidate action proposals for each instance and then perform boundary regression and action recognition on each proposal. These proposals can be generated using fixed anchors of single or multiple sizes [20–23] or through direct boundary regression [24, 25]. Although two-stage frameworks often incorporate intricate fusion techniques, they are prone to complicated design and error propagation from the proposal stage. Single-stage methods [26–29], on the other hand, directly predict temporal boundaries and action categories for each instance. Anchor-free methods [4, 30–33] perform action boundary regression without relying on default anchors. Due to their simple yet efficient frameworks, recent anchor-free models such as ActionFormer [4], TriDet [31], and MSTN [1] have achieved remarkable performance in the TAL task. At present, related detection technology has been widely applied in industrial scenarios [34, 35].

### Semi-supervised learning

Semi-Supervised Learning (SSL) methods [36] can generally be categorized into four types: generative methods [37, 38], graph-based methods [39, 40], consistency regularization methods [8, 41–45], and pseudo-labeling methods [46–49]. SSL finds extensive applications in visual tasks, including image classification, object detection, and semantic segmentation. Many SSL methods for these tasks utilize pseudo-label techniques [50–53] and the consistency learning framework [43, 44]. The Mean Teacher approach [8] averages model weights using Exponential Moving Average (EMA) to enhance performance without altering the network architecture. Active Teacher [54] evaluates unlabeled samples based on three key criteria, maximizing the utilization of limited label information. PseCo [55] introduces multi-view scale-invariant learning for object detection. Additionally, Zhang et al. [56] and PCL [57] aim to refine pseudo labels for more reliable training.

### Semi-supervised temporal action localization

Existing Semi-Supervised Temporal Action Localization (SS-TAL) methods can be classified into two main branches. The first branch centers on consistency learning and self-training [9, 58, 59]. KFC [58] demands that a single model make consistent predictions for features with added spatial and temporal perturbations. Ji et al. [59] subsequently integrate the teacher-student framework with SS-TAL, introducing time warping and time masking strategies. Furthermore, SSTAP [9] introduces a self-supervised module that learns relation-aware features through feature reconstruction and clip-order prediction training. The second branch of SS-TAL explores post-processing methods [5, 10]. SPOT [10] proposes a single-stage TAL solution to mitigate error propagation between action proposal generation and classification. NPL [5] combines class scores with boundary ambiguity and limits the maximum number of predictions per video, offering a different perspective on SS-TAL. These research efforts have achieved favorable results in experiments. However, methods utilizing pseudo labels have not adequately focused on the pseudo labels themselves, which contain valuable video knowledge. Therefore, we propose our SS-TAL framework, PLR, which emphasizes addressing the noise within pseudo labels. Our research on utilizing and improving pseudo labels aims to fill this gap in the field of SS-TAL.

## Methods

### Preliminary

#### Problem definition

The task of SS-TAL is to improve the performance of TAL network by fully utilizing both labeled videos ${\left\{X_{l}^{\left(i\right)}\right\}}_{i=1}^{N_{l}}$ and unlabeled videos ${\left\{X_{u}^{\left(i\right)}\right\}}_{i=1}^{N_{u}}$. Each action segment in the labeled video is annotated with *y*_*l*_ = ((*s*, *e*), *c*), where *s*, *e* and *c* refer to start time, end time, and category of this action instance. The performance of TAL network is reflected in its accuracy in temporal localization $\left(\hat{s},\hat{e}\right)$ and classification $\hat{c}$.

#### Video encoding

To compare fairly with other SS-TAL methods, we use the same pre-trained video encoders on certain detectors as other methods to align the data input. More specifically, we use two stream I3D [60] and TSN [61] to extract video features, which is consistent with NPL [5] and SPOT [10]. The pre-extracted features of a single video can be represented as **x**. The video encoders are not updated during training.

#### Teacher-student framework

The method presented is rooted in the Teacher-student framework [8]. This framework is characterized by two key aspects that synergistically enhance the learning process, particularly in semi-supervised learning scenarios.

Firstly, the teacher model is constructed as an Exponential Moving Average (EMA)-ensembled version of the student model. Mathematically, this can be expressed as:

$$\begin{array}{c} \begin{array}{c} \theta_{\text{teacher}}^{\left(t\right)}=\alpha \theta_{\text{teacher}}^{\left(t-1\right)}+\left(1-\alpha \right)\theta_{\text{student}}^{\left(t\right)}, \end{array} \end{array}$$ (1)

where *θ* represents the model parameters, *t* denotes the time step corresponding to each update of student weight, and *α* is the EMA decay rate. The teacher model maintains an inference mode. Given the superior performance of the ensembled model, the teacher is leveraged to generate pseudo-labels ${\hat{y}}_{u}$ using the features of unlabeled samples **x**_*u*_, which are then used to supervise the training of the student model. This process can be formulated as:

$$\begin{array}{c} \begin{array}{c} L_{student}=L\left(f_{\text{student}}\left({\text{x}}_{l}\right),y_{l}\right)+L\left(f_{\text{student}}\left({\text{x}}_{u}\right),{\hat{y}}_{u}\right), \end{array} \end{array}$$ (2)

where **x**_*l*_ and *y*_*l*_ represent features of labeled samples and their corresponding labels, L denotes the loss function. Specifically, we use IoU for localization loss and FocalLoss [62] for classification loss.

Secondly, a distinctive feature of this framework is the differential data exposure between the teacher and student models. The teacher model exclusively uses unlabeled data **x**_*u*_ without noise during inference, while the student model is trained on data that includes noise, denoted as **x**_*u*_ + *ψ*. This setup allows the student model to learn features that are robust to noise through the optimization constraints of consistent prediction. Specifically, the student model is encouraged to make predictions that are consistent with those of the teacher model, even when faced with noisy inputs. This robustness is then communicated back to the teacher model through the EMA updating mechanism, ensuring that the teacher also benefits from this noise-resistant learning.

### Overview

**Fig 2:**
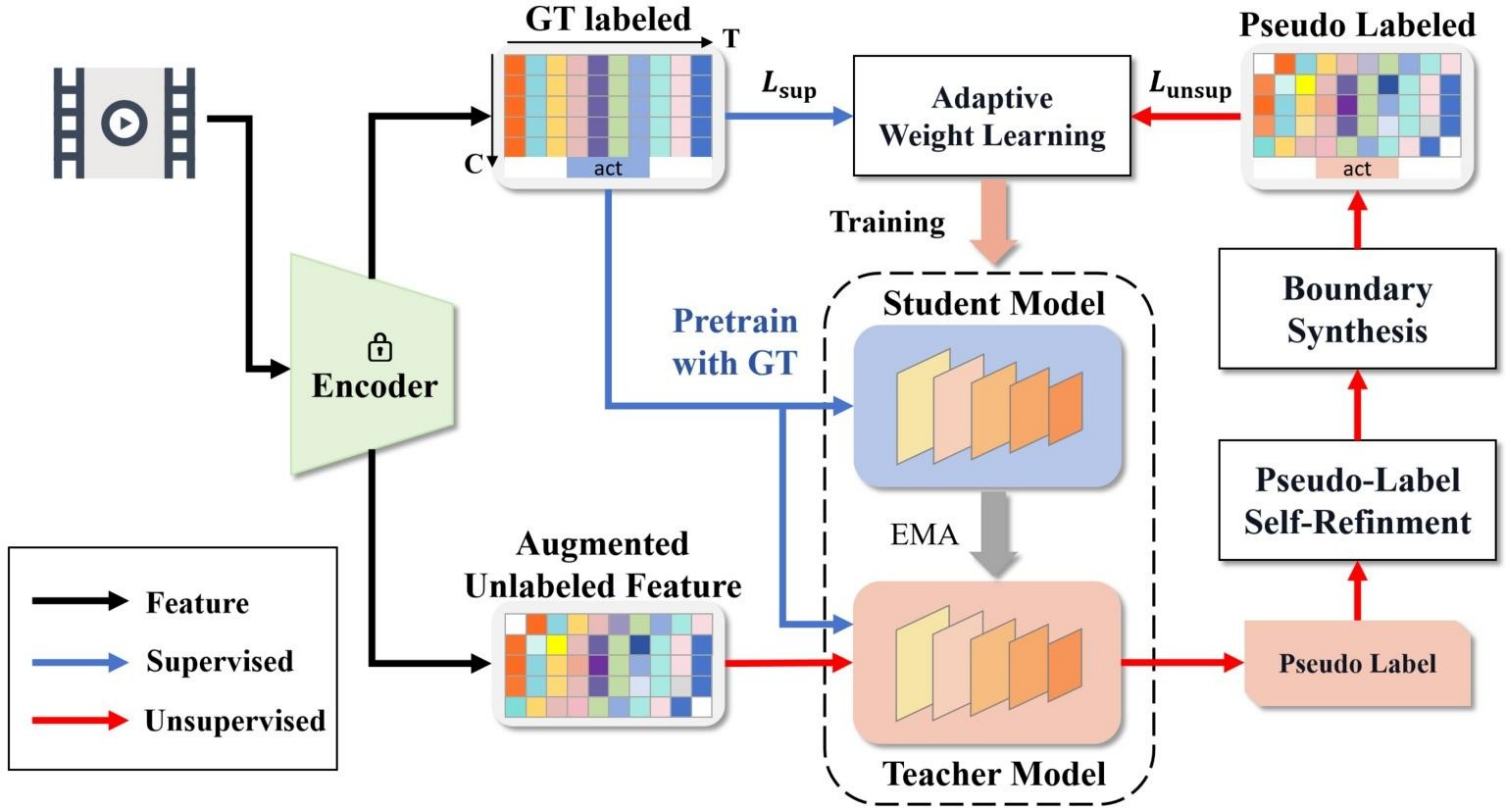
An overview of the pseudo label refining framework. In the feature extraction stage, labeled and unlabeled video features are obtained from the original video. In the pre-training stage, labeled features are used to pre-train the initial model. In the semi-supervised training stage, the initial model initializes the student and teacher models. The teacher model outputs initial pseudo-labels using unlabeled features, which are refined through self-refinement and boundary synthesis for subsequent student model training. The student model also trains on labeled data. Weight updates consider pseudo-label confidence.

We design a semi-supervised temporal action localization method based on teacher-student framework. The method consists of three parts: pseudo-label self-refinement (PLSR), boundary synthesis (BS), and adaptive weight learning (AWL). Before the start of training, the original videos are input to video encoder to obtain video features. Then, in the pre-training stage, the labeled video features are used to pre-train the initial model and PLSR module. Then, in the semi-supervised training stage, the initial model is used to initialize the student model and the teacher model. The unlabeled video features are input into the teacher model and the teacher model generates the initial pseudo labels. Then PLSR and BS are applied to refine the pseudo labels, which are used for training the student model. At the same time, the student model also receives training from labeled data. When updating weights according to loss, different weights are assigned based on the confidence of the pseudo-labels. The overall process of this method is shown in Fig 2.

### Pseudo-label self-refinement

#### Motivation

In object detection, Fast R-CNN [15] is a seminal work that incorporates a region proposal network (RPN) as well as feature extraction module, region of interest (ROI) pooling layer, classification module and boundary regression module, where boundary regressor plays the role of refining the proposal boxes:

$$\begin{array}{c} \begin{array}{c} b=\text{Regressor}(\text{ROI}(\text{z},p)), \end{array} \end{array}$$ (3)

where $\text{z}\in R^{C\times H\times W}$ denotes the feature map of the entire image, *p* refers to proposal generated by RPN, and $b\in R^{1\times 4}$ is the predicted bounding box. This process involves extracting features from the proposed region using the convolutional layers of the neural network, followed by applying fully connected layers to predict the coordinate offsets of the bounding box. Regressor itself implements fine-tuning of proposals, and can obviously also be used for fine-tuning of bounding boxes. Simply input the predicted bounding box *b* as a proposal to the latter half of neural networks:

$$\begin{array}{c} \begin{array}{c} b_{\text{refined}}=\text{Regressor}\left(\text{ROI}\left(\text{z},b\right)\right), \end{array} \end{array}$$ (4)

Where *b*_refined_ denotes the refined bounding box, and feature map **z** is reused.

In temporal action localization, the predicted action temporal boundary $I=\left(s,e\right)\in R^{1\times 2}$ is a one-dimensional boundary, and the initial 2D regression structure is no longer applicable. To solve this problem, a pseudo-label self-refinement module (PLSR) tailored for 1D temporal boundary is proposed.

#### Implementation details of PLSR

In temporal action localization, the pre-extracted features of a single video can be represented as **x**, and it will be processed by the backbone of detector to generate more latent representation $\text{z}\in R^{C\times T}$ where *C* represents the feature dimension and *T* represents the temporal dimension. Initially, the latent representation **z**_*s*:*e*_ corresponding to the temporal interval *I* = (*s*, *e*) is selected. Then, linear interpolation is applied along the temporal dimension to resize it into a fixed-size representation ${\text{z}}^{\prime }\in R^{C\times T^{\prime }}$. In this method, *T*′ is set according to the distribution of action instance durations.

Due to the difference of tasks, the ROI pooling layer applied to the two-dimensional feature map is replaced with a one-dimensional convolutional layer with a convolution kernel. After this step, a deeper representation ${\text{z}}^{\prime \prime }\in R^{C\times T^{\prime \prime }}$ can be obtained:

$$\begin{array}{c} \begin{array}{c} {\text{z}}^{\prime }=\text{Interpolation}\left({\text{z}}_{s:e}\right),\\ {\text{z}}^{\prime \prime }=\text{Conv1d}\left({\text{z}}^{\prime }\right), \end{array} \end{array}$$ (5)

Input **z**″ into the classifier and regressor of the original detector to obtain the classification prediction confidence $f_{c}\left({\text{z}}^{\prime \prime }\right)\in R^{N_{\text{cls}}\times T^{\prime \prime }}$ and interval offset $f_{r}\left({\text{z}}^{\prime \prime }\right)\in R^{2\times T^{\prime \prime }}$, where *N*_cls_ represents the number of action types in the action set **C**. Input *f*_*c*_(**z**″) and *f*_*r*_(**z**″) into the corresponding two fully connected layers to obtain the class-wise prediction confidence score $\hat{\text{c}}\in R^{N_{\text{cls}}\times 1}$ and interval offset $\hat{\text{r}}\in R^{2\times 1}$. This process can be represented as follows:

$$\begin{array}{c} \begin{array}{c} \hat{\text{c}}={\text{FC}}_{c2}\left({\text{FC}}_{c1}\left(f_{c}\left({\text{z}}^{\prime \prime }\right)\right)\right),\\ \hat{\text{r}}={\text{FC}}_{r2}\left({\text{FC}}_{r1}\left(f_{r}\left({\text{z}}^{\prime \prime }\right)\right)\right), \end{array} \end{array}$$ (6)

Further, take the maximum value of $\hat{\text{c}}$ as the classification confidence, and add $\hat{\text{r}}$ to the original *I* to obtain the fine-tuned temporal boundary:

$$\begin{array}{c} \begin{array}{c} \hat{c}=\text{sigmoid}\left(\text{max}\left(\hat{\text{c}}\right)\right),\\ I_{\text{refined}}=I+\hat{\text{r}}, \end{array} \end{array}$$ (7)

**Fig 3:**
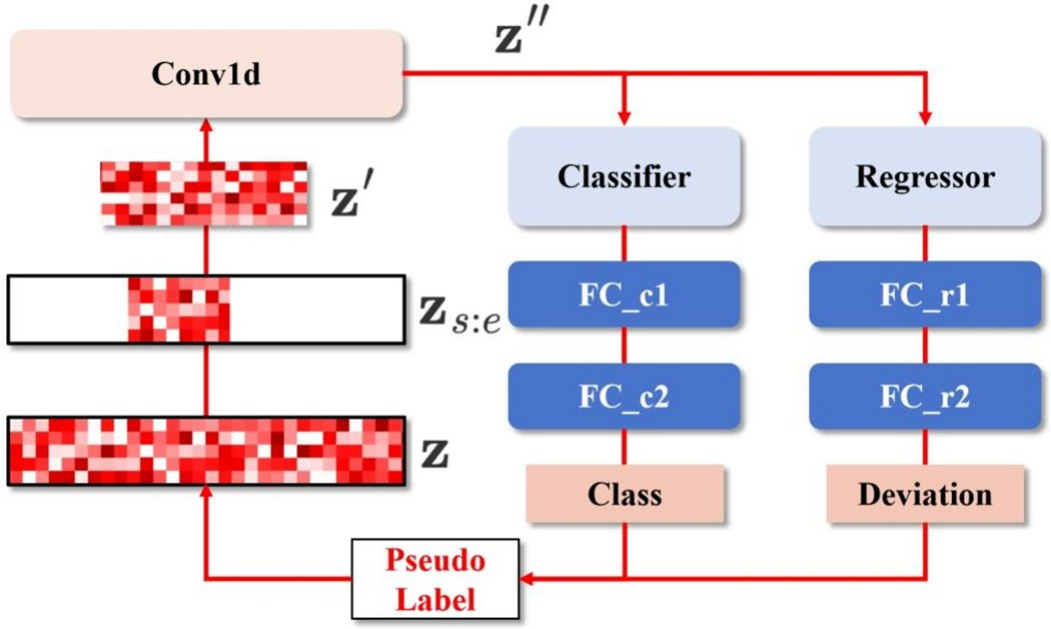
The illustration of pseudo label self refinement module. Re-scaled and pooled representation **Z**″ is fed into the classifier and regressor to obtain prediction confidence and interval offsets, processed through fully connected layers, and used to obtain the fine-tuned temporal boundary.

Now we define ${\hat{y}}^{\left(0\right)}=\left(I_{\text{refined}}^{\left(0\right)},{\hat{c}}^{\left(0\right)}\right)$. The above process can be referred to Fig 3.

### Boundary synthesis

#### Motivation

The latent representation of a single video mentioned above can be represented as a tensor of $\text{z}\in R^{C\times T}$. Based on this, the classifier and regressor of the detector will perform *T* times of intensive predictions, including action category prediction and action interval prediction. Then, based on the confidence scores of different predicted categories, Non-Maximum Suppression (NMS) or SoftNMS is used for post-processing to obtain the final prediction. This means that the prediction with the highest score (maximum prediction) will make other predictions near it invisible, but due to the inaccuracy of the pseudo label, some inaccurate predictions will be given high confidence.

To mitigate the negative impact of this problem, it is necessary to make full use of the information from the context of maximum prediction. Based on pseudo-label self-refinement, a boundary synthesis mechanism based on multi-vote weight summation is proposed.

#### Implementation details of BS

**Fig 4:**
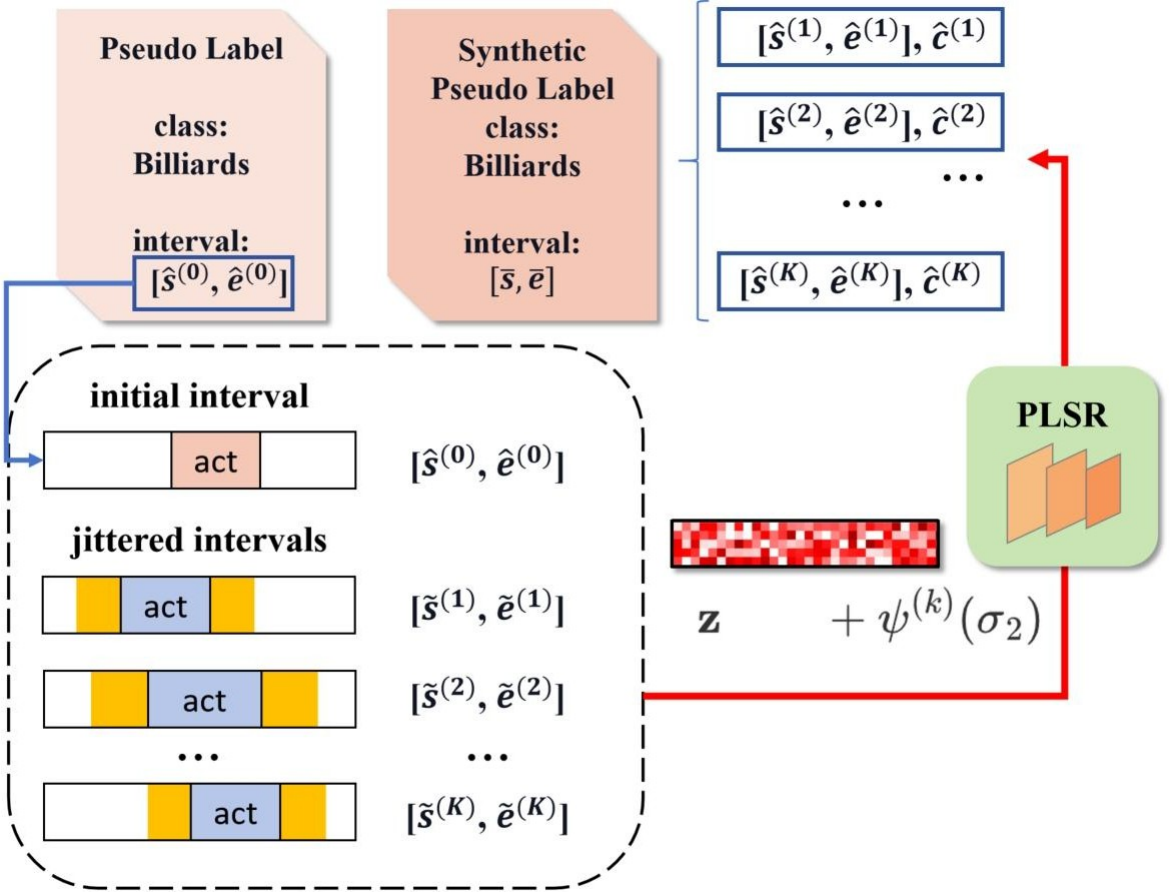
The illustration of boundary synthesis module. It improves accuracy of pseudo labels by leveraging information from the context of maximum prediction ${\hat{y}}^{\left(0\right)}=\left(I_{\text{refined}}^{\left(0\right)},{\hat{c}}^{\left(0\right)}\right)$ through three steps: random boundary disturbance, adding Gaussian noise to corresponding features, and averaging multiple predictions weighted by their confidence scores.

The process is divided into three steps, as shown in Fig 4.

The first step is to randomly add noise to the existing action intervals $I_{\text{refined}}^{\left(0\right)}=\left({\hat{s}}^{\left(0\right)},{\hat{e}}^{\left(0\right)}\right)$ to obtain new action intervals, and then expand the intervals by 0.5 times the length to obtain expanded intervals:

$$\begin{array}{c} \begin{array}{c} \tilde{s}={\hat{s}}^{\left(0\right)}+\left(\psi_{s}-0.5\right)\left({\hat{e}}^{\left(0\right)}-{\hat{s}}^{\left(0\right)}\right),\psi_{s}∼N\left(0,\sigma_{1}\right),\\ \tilde{e}={\hat{e}}^{\left(0\right)}+\left(\psi_{e}+0.5\right)\left({\hat{e}}^{\left(0\right)}-{\hat{s}}^{\left(0\right)}\right),\psi_{e}∼N\left(0,\sigma_{1}\right), \end{array} \end{array}$$ (8)

where *σ*_1_ is the hyperparameter to be set. This step repeats *K* times to get ${\left\{{\tilde{y}}^{\left(k\right)}=\left(\left({\tilde{s}}^{\left(k\right)},{\tilde{e}}^{\left(k\right)}\right),{\hat{c}}^{\left(0\right)}\right)\right\}}_{k=1}^{K}$.

The second step is to select the features corresponding to these expanded intervals and add Gaussian noise to them:

$$\begin{array}{c} \begin{array}{c} {\tilde{\text{z}}}^{\left(k\right)}=\text{z}+\psi_{z},\psi_{z}∼N\left(0,\sigma_{2}\right), \end{array} \end{array}$$ (9)

where *σ*_2_ is the hyperparameter to be set. This process can also be written as ${\tilde{\text{z}}}^{\left(k\right)}=\text{z}+\psi^{\left(k\right)}\left(\sigma_{2}\right)$.

The third step is to infer the features of these specific intervals with noise:

$$\begin{array}{c} \begin{array}{c} I_{\text{refined}}^{\left(k\right)},{\hat{c}}^{\left(k\right)}=f_{\text{PLSR}}\left({\tilde{\text{z}}}^{\left(k\right)},{\tilde{y}}^{\left(k\right)}\right), \end{array} \end{array}$$ (10)

where *k* ∈ [1, *K*]. This process can also be written as ${\hat{y}}^{\left(k\right)}=f_{\text{PLSR}}\left(\text{z}+\psi^{\left(k\right)}\left(\sigma_{2}\right),{\tilde{y}}^{\left(k\right)}\right)$.

Finally, average the inference results according to confidence score:

$$\begin{array}{c} \begin{array}{c} \bar{I}=\frac{1}{\sum_{k=1}^{K}{\hat{c}}^{\left(k\right)}}\sum_{k=1}^{K}{\hat{c}}^{\left(k\right)}I_{\text{refined}}^{\left(k\right)}, \end{array} \end{array}$$ (11)

notice that the class-wise prediction confidence score ${\hat{c}}^{\left(k\right)}$ is retained for the following part.

### Adative weight learning

#### Motivation

The learning difficulty of different unlabeled samples varies significantly, with some being easier to learn from than others. Additionally, the unbalanced distribution of unlabeled samples further complicates the issue, as it results in pseudo labels generated by the teacher model being of unequal reliability.

To address this phenomenon and ensure that the learning process is not unduly influenced by unreliable pseudo labels, a dynamic weight is designed that comprehensively considers the credibility of each pseudo label. This approach allows the model to adaptively adjust the importance of each pseudo label based on its reliability, thereby improving the overall performance and robustness of the TAL model.

#### Implementation details of AWL

Here, we use mean information entropy (mIE) to measure the pseudo label of certain action instance:

$$\begin{array}{c} \begin{array}{c} mIE=-\frac{1}{KC}\sum_{k=1}^{K}\sum_{c=1}^{C}{\hat{c}}_{c}^{\left(k\right)}\;\text{log}\;{\hat{c}}_{c}^{\left(k\right)}, \end{array} \end{array}$$ (12)

where *K* here is same as the one in BS, and *C* denotes the class number. When the output corresponding to the classification is more evenly distributed, the mIE is higher, and the corresponding pseudo-labels are less reliable. To give a simple example, when the classification output is entirely 0 or 1, the mIE is 0; in other cases, the mIE value is greater than 0.

Our approach is to initially focus on learning from high-confidence pseudo labels and gradually increase the weight of low-confidence pseudo labels, loss weight for each epoch is calculated by:

$$\begin{array}{c} \begin{array}{c} w_{\text{unsupervised}}=\frac{2}{e}-\left(1-\frac{epoch}{0.5*epoch_{\text{max}}}\right)mIE, \end{array} \end{array}$$ (13)

where *epoch* represents the training round, and *epoch*_max_ represents the maximum number of training rounds. *w*_unsupervised_ is used as a factor multiplied by the loss calculated by pseudo labels. At the initial stage of training, the coefficient in front of mIE is -1, meaning that pseudo-labels with higher mIE (i.e., lower confidence) have smaller weights, and conversely, pseudo-labels with higher confidence have larger weights. As training approaches its end, the coefficient in front of mIE becomes 1, meaning that pseudo-labels with higher mIE (i.e., lower confidence) have larger weights, and conversely, pseudo-labels with higher confidence have smaller weights. This way, we can prioritize the learning from more reliable labels while still incorporating information from less reliable ones, ultimately improving the overall performance and robustness of the model.

### Training process

In this section, we describe the training strategy we devised. To provide a more intuitive explanation of our method, we present the training pseudocode in Algorithm 1. The first training stage involves training a model and PLSR using only labeled data, and initializing both the teacher model and the student model with this trained model. In the second training stage, at the beginning of each epoch, the teacher model combined with PLSR and BS is used to generate pseudo-labels for all unlabeled data. During this process, the parameters of the PLSR component continue to be trained with labeled data, while the teacher model is not updated. These pseudo-labels are then used to train the student model, which subsequently updates the weight of the student model to the teacher model via Exponential Moving Average (EMA).

**Algorithm 1** Training process of the proposed PLR

**Require**: Training dataset *D* = {(**x**_*l*_, **x**_*u*_, *y*_*l*_)} and hyperparameters: *α*, *K*, *σ*_1_, *σ*_2_

 1: Initialize model parameters *θ*_model_: (*θ*_backbone_, *θ*_head_), *θ*_PLSR_.

        ▹ Stage 1: Supervised Pre-training

 2: **for** each training iteration **do**

 3:  Sample a batch *B* = {(**x**_*l*_, *y*_*l*_)} from *D*

 4:  **z**_*l*_ ← *f*_backbone_(**x**_*l*_)

 5:  ${\hat{y}}_{l}\leftarrow f_{\text{head}}\left({\text{z}}_{l}\right)$

 6:  ${\hat{y}}_{l}^{\left(0\right)}\leftarrow f_{\text{PLSR}}\left(\text{detach}\left({\text{z}}_{l}\right),{\hat{y}}_{l}\right)$

 7:  $L_{\text{model}}\leftarrow L\left({\hat{y}}_{l},y_{l}\right)$

 8:  $L_{\text{PLSR}}\leftarrow L\left({\hat{y}}_{l}^{\left(0\right)},y_{l}\right)$

 9:  UpdateParameters($\theta_{\text{model}},L_{\text{model}}$)

 10:  UpdateParameters($\theta_{\text{PLSR}},L_{\text{PLSR}}$)

 11: **end for**

         ▹ Stage 2: Semi-Supervised Training

 12: *θ*_student_(train), *θ*_teacher_(eval) ← *θ*_model_

 13: **for** each training iteration **do**

 14:  Sample a batch *B* = {(**x**_*l*_, **x**_*u*_, *y*_*l*_)} from *D*

          ▹ Supervised Part

 15:  **z**_*l*_ ← *f*_student_backbone_(**x**_*l*_)

 16:  ${\hat{y}}_{l}\leftarrow f_{\text{student\_head}}\left({\text{z}}_{l}\right)$

 17:  ${\hat{y}}_{l}^{\left(0\right)}\leftarrow f_{\text{PLSR}}\left(\text{detach}\left({\text{z}}_{l}\right),{\hat{y}}_{l}\right)$

 18:  $L_{\text{supervised}}\leftarrow L\left({\hat{y}}_{l},y_{l}\right)$

 19:  $L_{\text{PLSR}}\leftarrow L\left({\hat{y}}_{l}^{\left(0\right)},y_{l}\right)$

       ▹ Unsupervised Part

 20:  **z**_*u*_ ← *f*_teacher_backbone_(**x**_*u*_)

 21:  ${\hat{y}}_{u}\leftarrow f_{\text{teacher\_head}}\left({\text{z}}_{u}\right)$

 22:  ${\hat{y}}_{u}^{\left(0\right)}\leftarrow f_{\text{PLSR}}\left({\text{z}}_{u},{\hat{y}}_{u}\right)$

 23:  ${\left\{{\tilde{y}}_{u}^{\left(k\right)}\right\}}_{k=1}^{K}\leftarrow \text{RandomBoundaryExpanding}\left({\hat{y}}_{u}^{\left(0\right)},K,\sigma_{1}\right)$

 24:  $preds\leftarrow [\text{detach}\left({\hat{y}}_{u}^{\left(0\right)}\right),\;]$

 25:  **for** *k* from 1 to *K* **do**

 26:   ${\hat{y}}_{u}^{\left(k\right)}\leftarrow f_{\text{PLSR}}\left({\text{z}}_{u}+\psi^{\left(k\right)}\left(\sigma_{2}\right),{\tilde{y}}_{u}^{\left(k\right)}\right)$

 27:   $\text{Append}(preds,\text{detach}\left({\hat{y}}_{u}^{\left(k\right)}\right))$

 28:  **end for**

 29:  ${\bar{y}}_{u}\leftarrow \text{SynthesisByScore}\left(preds\right)$

 30:  $L_{\text{unsupervised}}\leftarrow L\left(f_{\text{student}}\left({\text{x}}_{l}+\psi \left(\sigma_{2}\right)\right),{\bar{y}}_{u}\right)$

          ▹ Update Parameters

 31:  UpdateParameters($\theta_{\text{student}},L_{\text{supervised}}+L_{\text{unsupervised}}$)

 32:  UpdateParameters($\theta_{\text{PLSR}},L_{\text{PLSR}}$)

 33:  *θ*_teacher_ ← *αθ*_teacher_ + (1 − *α*)*θ*_student_

 34: **end for**

## Results and discussion

### Dataset

We evaluate our methods on two TAL benchmark datasets, THUMOS14 [11] and ActivityNet v1.3 [12]. THUMOS14 [11] provides 1010 videos for validation, with 220 temporally annotated videos, and 1574 videos for testing, with 212 temporally annotated videos from 20 action categories. Following common practice, the proposed network is trained on the validation set and evaluated on the test set. The ActivityNet-v1.3 [12] is classified into training, validation, and test subsets, which contain 10024, 4926, and 5044 videos from 200 categories, respectively. Following the standard evaluation protocol, the network is trained on the test subset, and the evaluation results are reported on the validation subset.

For semi-supervised task, randomly select 10%, 20%, 40%, and 60% of samples from each action category as labeled samples, and the remaining samples are considered unlabeled samples. Our experiment is conducted at these four label rates.

### Implementation details

We use ActionFormer [4] and BMN [17] as the basic TAL model for our method, following NPL [5].

When employing ActionFormer as the base detector, in the pre-training stage, we only use labeled data to pretrain for 40/15 epochs(for THUMOS14 and ActivityNet v1.3 respectively), the learning rate is set to 1e-4/1e-3, and the learning rate strategy includes 10/5 epochs of warmup and 30/10 epochs of cosine annealing, weight decay is set to 0.05, EMA *α* is set to 0.995 for the 10% label rate and 0.999 for the 60% label rate. In the semi-supervised stage, we utilize both labeled and unlabeled data for training, and set EMA *α* to 0.999. The remaining training settings remain identical to those used in the pre-training stage.

When using BMN as the base detector, in the pre-training stage, we use labeled data to pretrain for 30/30 epochs with a learning rate of 1e-3/4e-3 and a weight decay of 5e-3/5e-3. Further, we improve the model with pseudo labels for 15 epochs with EMA *α* been set to 0.99. The temporal dimension of BMN is set to 100/256 for ActivityNet/THUMOS14 respectively.

For key hyper-parameters, *K* is set to 4, along with *σ*_1_ set to 0.05 and *σ*_2_ set to 0.01. For data augmentation, we apply a 10% time mask, Gaussian sampled temporal scaling. All pseudo labels are filtered based on a class-confidence threshold of 0.2. Besides, SoftNMS is employed for post-processing and experiments are conducted on RTX 2080 Ti × 1 with fixed random seed.

### Comparison with other methods

We compare the proposed method PLR with existing main SS-TAL methods in Table 1.

**Table 1:** Performance comparison with state-of-the-art methods on THUMOS14 and ActivityNet v1.3 with ActionFormer(ActF) and BMN.

Label	Method	Feature	THUMOS14(%)						ActivityNet v1.3(%)			
			0.3	0.4	0.5	0.6	0.7	Avg.	0.5	0.75	0.95	Avg.
10%	ActF [4]	I3D	28.5	22.9	14.1	8.2	4.1	15.6	47.8	24.2	1.7	25.6
	ActF [4] + MixUp [63]	I3D	29.7	24.2	14.5	9.6	5.4	16.7	49.7	27.9	3.1	28.8
	ActF [4] + NPL [5]	I3D	32.8	29.6	20.1	11.7	7.2	20.3	51.9	33.4	3.6	32.5
	**ActF** [4] + **PLR(Ours)**	I3D	**34.6**	**31.2**	**23.0**	**13.4**	**9.2**	**22.3**	**52.2**	**33.6**	**4.1**	**32.9**
	SSP [59]	TSN	44.2	34.1	24.6	16.9	9.3	25.8	38.9	28.7	8.4	27.6
	SPOT [10]	TSN	49.4	40.4	31.5	22.9	12.4	31.3	49.9	31.1	8.3	32.1
	BMN [17] + SSTAP [9]	TSN	45.6	35.2	26.3	17.5	10.7	27.0	40.7	29.6	**9.0**	28.2
	BMN [17] + NPL [5]	TSN	50.0	41.7	33.5	23.6	13.4	32.4	50.9	32.0	7.9	32.6
	**BMN** [17] + **PLR(Ours)**	TSN	**50.5**	**42.2**	**34.0**	**24.2**	**13.4**	**32.9**	**52.4**	**32.6**	7.9	**33.1**
20%	ActF [4]	I3D	49.1	41.6	32.6	21.5	12.1	31.4	51.2	34.3	3.8	32.9
	ActF [4] + MixUp [63]	I3D	51.2	43.2	34.0	23.9	14.1	33.3	52.9	34.7	3.9	33.3
	ActF [4] + NPL [5]	I3D	54.5	47.1	39.3	29.7	18.5	37.8	53.1	35.8	3.9	33.8
	**ActF** [4] + **PLR(Ours)**	I3D	**57.1**	**50.8**	**42.8**	**30.4**	**19.5**	**40.1**	**54.1**	**36.7**	**4.4**	**34.3**
	SPOT [10]	TSN	52.6	43.9	34.1	25.2	16.2	34.4	51.7	32.0	6.9	32.3
	BMN [17] + NPL [5]	TSN	53.9	45.6	36.2	26.9	16.5	35.8	52.1	32.9	7.9	32.9
	**BMN** [17] + **PLR(Ours)**	TSN	**54.5**	**46.5**	**37.3**	**28.3**	**18.4**	**37.0**	**52.9**	**33.5**	**8.4**	**33.7**
40%	ActF [4]	I3D	69.0	60.4	49.3	31.5	19.3	45.9	53.2	35.7	3.8	34.2
	ActF [4] + MixUp [63]	I3D	69.7	61.9	52.4	34.4	20.1	47.7	53.1	36.0	4.3	34.5
	ActF [4] + NPL [5]	I3D	71.9	65.4	55.7	40.9	23.4	51.5	53.6	36.5	4.6	35.3
	**ActF** [4] + **PLR(Ours)**	I3D	**72.7**	**66.9**	**58.5**	**45.8**	**31.0**	**55.0**	**54.1**	**36.7**	**6.2**	**35.6**
	SPOT [10]	TSN	54.4	45.8	37.2	29.7	19.4	37.3	53.3	33.0	6.6	33.2
	BMN [17] + NPL [5]	TSN	56.2	46.7	38.8	30.3	19.5	38.3	53.4	33.9	8.1	33.8
	**BMN** [17] + **PLR(Ours)**	TSN	**56.9**	**47.9**	**39.5**	**31.3**	**20.3**	**39.2**	**53.7**	**34.4**	**8.5**	**34.5**
60%	ActF [4]	I3D	71.5	65.6	59.9	47.3	32.7	55.4	53.9	36.1	5.7	35.0
	ActF [4] + MixUp [63]	I3D	72.2	67.5	61.2	48.7	34.0	56.7	54.1	36.4	5.7	35.2
	ActF [4] + NPL [5]	I3D	74.5	69.9	62.8	51.1	36.6	59.0	54.3	36.7	6.5	35.8
	**ActF** [4] + **PLR(Ours)**	I3D	**78.5**	**74.0**	**64.7**	**51.8**	**37.0**	**61.2**	**54.5**	**36.7**	**8.2**	**36.0**
	SSP [59]	TSN	53.2	46.8	39.3	29.7	19.8	37.8	49.8	34.5	7.0	33.5
	SPOT [10]	TSN	58.9	50.1	42.3	33.5	22.9	41.5	52.8	35.0	8.1	35.2
	BMN [17] + SSTAP [9]	TSN	56.4	49.5	41.0	30.9	21.6	39.9	50.1	34.9	7.4	34.0
	BMN [17] + NPL [5]	TSN	59.0	51.4	42.9	34.3	23.3	42.2	53.9	35.8	8.5	35.7
	**BMN** [17] + **PLR(Ours)**	TSN	**60.3**	**52.2**	**43.9**	**34.6**	**23.6**	**42.9**	**54.7**	**36.4**	**8.5**	**35.9**

The table compares the performance of our proposed PLR method with several state-of-the-art approaches on the THUMOS14 and ActivityNet v1.3 datasets. The results are presented in terms of average precision(AP) and mean average precision(mAP). For THUMOS14 [11], thresholds of IoU are [0.3:0.7:0.1]. And for ActivityNet v1.3, thresholds of IoU are [0.5:0.95:0.05], we report three sets of AP and mAP as others do.

On THUMOS14 [11], our PLR method, when combined with ActionFormer [4] and BMN [17], achieves the highest mAP of 61.2% and 42.9% at 60% label rate, outperforming other methods such as MixUp [63], NPL [5], and SPOT [10]. Similarly, at other label rates, PLR surpasses other SOTA methods as well.

On ActivityNet v1.3 [12], the PLR method consistently improves the performance of the base models, ActF and BMN, across different thresholds and datasets.

Overall, the results demonstrate the effectiveness of our PLR method in enhancing the performance of existing TAL models, achieving state-of-the-art results on both THUMOS14 and ActivityNet v1.3 datasets.

### Ablation and discussion

To test the effectiveness of each module in our proposed method, we design the following ablation experiments where ActionFormer [4] works as action detector with I3D [60] features.

#### Effectiveness of individual modules

We started with training using only pseudo labels and gradually added various modules to observe the effect of each module. Experiments are conducted at two tag rates of 10% and 60%, as results can be seen in Table 2. After adding pseudo label self-refinement(PLSR) separately, the mAP increased by 3.9% and 3.2%. When applying boundary synthesis(BS), the mAP increased by 4.0% and 3.7%. With adopting adaptive weight combined with PLSR and BS in training process getting corrected, the mAP goes to 22.3% and 61.2%.

**Table 2:** Ablation study on effectiveness of each component of the PLR on THUMOS14.

PLSR	BS	AWL	10%	60%
			15.6(+0.0)	55.4(+0.0)
✓			19.5(+3.9)	58.2(+3.2)
	✓		19.6(+4.0)	59.1(+3.7)
		✓	18.3(+2.7)	57.4(+2.0)
✓	✓	✓	**22.3(+6.6)**	**61.2(+5.8)**

We use 10% and 60% labeled videos for ablation experiments. The baseline is ActionFormer trained with vanilla pseudo label.

#### Effectiveness of pseudo-label self-refinement

The PLSR module is specifically designed to refine the temporal boundaries of the pseudo labels, aiming to enhance the precision of these labels in terms of their temporal alignment. To thoroughly evaluate its performance, we examine it from an Intersection over Union (IoU) perspective. This metric allows us to quantify the degree of overlap between the predicted temporal boundaries and the ground-truth boundaries. Consequently, the effectiveness of the PLSR module’s boundary correction can be accurately reflected by the mean IoU score calculated between the predictions and the ground-truths. A higher mean IoU indicates better boundary refinement, demonstrating the module’s capability to improve the accuracy of pseudo labels in temporal localization tasks.

As shown in Table 3, model trained with refined pseudo labels exhibit better localization ability compared to model trained with vanilla pseudo labels, which demonstrates the effectiveness of PLSR. At a label rate of 10%, using fine-tuned pseudo labels, the average IoU and accuracy improved by 0.05 compared to before fine-tuning, which also increased the mAP by 3.9%. The same was true at a label rate of 60%, where the average IoU of pseudo labels improved by 0.08 compared to before, resulting in a 2.8% increase in accuracy.

**Table 3:** Ablation study on effectiveness of PLSR.

Method	10%		60%	
	tIoU	mAP	tIoU	mAP
baseline	0.189	15.6	0.293	55.4
**baseline + PLSR**	**0.241**	**19.5**	**0.377**	**58.2**

The model uses ActionFormer pre-trained with 40 epochs on labeled THUMOS14 data. The baseline is detector using vanilla pseudo label.

#### Effectiveness of boundary synthesis

The role of boundary synthesis(BS) is to reduce the inherent bias of the model through multiple inferences, which is similar to the specific application of TTA in TAL tasks during inference. BS consists of three main components: random boundary disturbance, adding Gaussian noise during inference, and boundary aggregation. We have separately tested the effectiveness of random boundary disturbance and Gaussian noise, as results reported in Table 4.

**Table 4:** Ablation study on effectiveness of BS component.

Method	10%	60%
	Avg. mAP(%)	Avg. mAP(%)
baseline	15.6	55.4
baseline + random boundary disturbance	17.8	57.7
baseline + Gaussian noise	19.1	58.5
**baseline + BS**	**19.6**	**59.1**

The model uses ActionFormer pre-trained with 40 epochs on labeled THUMOS14 data. The baseline is detector using vanilla pseudo label.

It should be notice that “+ Gaussian noise” represents using the exact same feature interval. It can be seen that Gaussian noise plays a key role in multiple inferences, and the model reduces the expected bias in multiple inferences. At the same time, random boundary disturbance makes this optimization effect better.

#### Impact of random seeds

**Fig 5:**
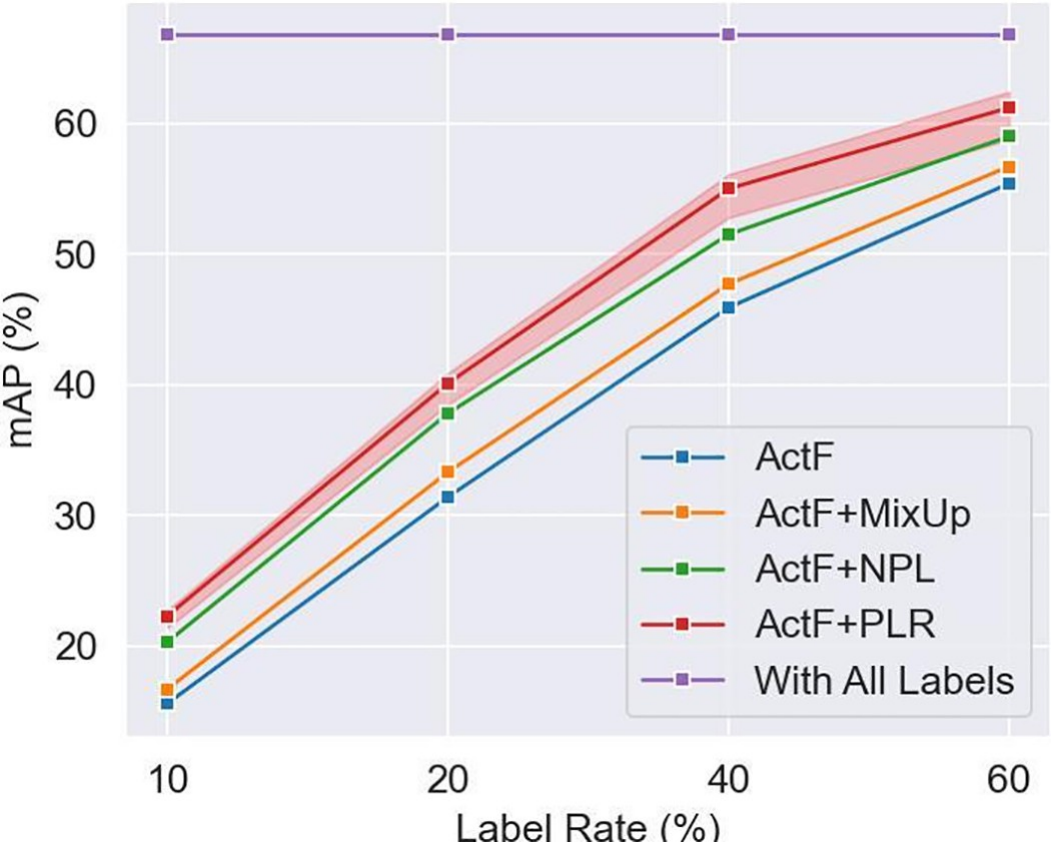
Averaged performance curve on THUMOS14.

We select 10 random seeds for a fixed set of settings to observe the impact of random seeds. We find that random seeds can cause the final mAP to fluctuate by up to 0.5%. However, our overall improvement is significantly higher than previous SOTA, thus indirectly reflecting the effectiveness of the PLR method. The averaged performance curve is shown in Fig 5.

### Inference speed

**Fig 6:**
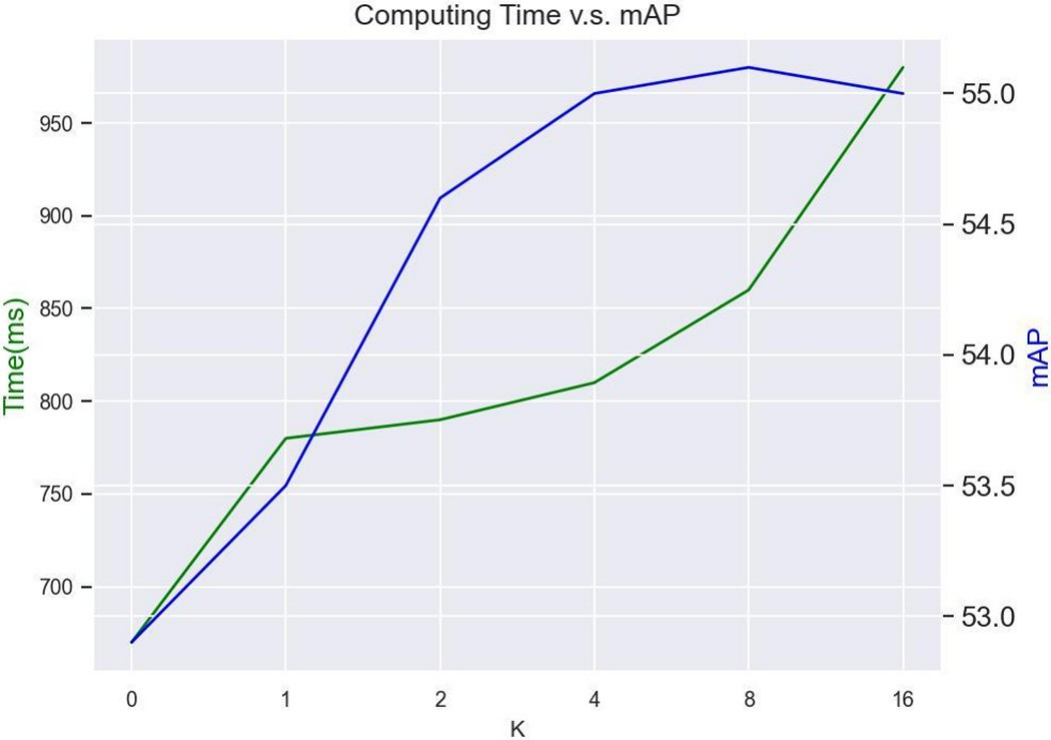
Trade-off between computing consumption brought by *K* and performance gain.

Although our proposed method significantly improves the quality of pseudo-labels, it requires more training time compared to conventional semi-supervised methods. Specifically, with *K* = 4 in this paper, it necessitates an additional 6 iterations of PLSR module inference compared to the most basic pseudo-labeling approach. To address this issue, we approach it from two perspectives: first, optimizing the code workflow to increase computational parallelism; second, selecting the most cost-effective value of *K*. We have changed the *K* iterations of inference to a single inference with the batch size increased by a factor of *K*. By optimizing the code workflow, we have parallelized the *K* iterations of the PLSR process, greatly reducing the extra inference overhead. According to our tests, processing one batch of samples through ActionFormer takes approximately 450ms, including pseudo label generation. While processing through PLSR takes about 110ms. If 6 additional iterations of PLSR module inference are required, the inference time for a single batch increases to 1120ms, representing a 148% increase over the original time. After parallelizing the K iterations of PLSR, the inference time for a single batch is reduced to 810ms, resulting in a 80%(68% less) increase compared to the vanilla pseudo label method. We conducted a search test for the parameter *K*, and below are the results for ActionFormer on the THUMOS14 dataset with a 40% labeling rate, as shown in Fig 6. We kept PLSR and AWL constant while varying the value of *K*. When *K* = 0, it means not using BS, resulting in two PLSR forward passes; when *K* = *N*(*N* > 0), it results in two PLSR inferences plus one large-batch parallelized PLSR forward pass. The test results are as follows: when *K* < = 4, the additional computational overhead is relatively small, and there is a significant performance improvement. When *K* > 4, there is no notable performance gain, but it introduces additional overhead. Based on these experimental results, we ultimately determined *K* to be 4.

#### Additional training time, taking THUMOS14 + ActionFormer as an example

In practical applications, using the model trained by our method for inference does not introduce any additional inference time, adhering to the original inference paradigm of the model. In other words, the extra time consumption associated with our method is introduced during the teacher-student framework training process. If only the training time is considered, without taking the inference scenario into account, the additional overhead incurred by our method is entirely acceptable, as can be seen in Table 5. Taking THUMOS14 as an example, extracting features from all these videos alone requires over 100 hours (thanks to previous researchers, as practical video data does not come with pre-extracted features), while our overall training time increases from 60 minutes with only standard pseudo-labels to approximately 100 minutes with our new method. In terms of training, the additional overhead of our method is less than 1%, yet it delivers significant performance improvements.

**Table 5:** Time consumption of training on 40% labeled THUMOS14 when our method is combined with ActionFormer.

Phase	Time Consumption(min)	Ratio(%)
Video Encoding	> 6000	> 98
Supervised Training	∼ 30	< 1
Vanilla Semi-supervised Training	∼ 60	< 1
**PLR Semi-supervised Training**	∼ **100**	< **2**

## Conclusion

This study proposes a pseudo-label refining method based on the semi-supervised teacher-student framework. It involves pseudo-label self-refinement, boundary synthesis and adaptive weight learning. Using ActionFormer [4] and BMN [17] as the detector, the method achieves significant improvement on the THUMOS14 [11] and ActivityNet v1.3 [12] dataset, outperforming other SSTAL methods in mAP at label rates of 10% to 60%. Ablation experiments demonstrate the effectiveness of each module in enhancing pseudo-label accuracy. The method proposed in this paper improves the accuracy of pseudo-labels by introducing new modules and increasing computational efforts. We believe that future research directions for SS-TAL can be explored in the following ways:

1. Better module design to provide a fine-grained mechanism for pseudo-label refinement, this has been preliminarily studied in SPOT [10];
2. In-depth investigation of the training process, as there is currently a lack of comprehensive discussion on training procedures under the same benchmark;
3. More reliable evaluation metrics for pseudo-labels. Currently, in SS-TAL, NPL [5] offers a pseudo-label evaluation metric that incorporates boundary confidence, but we believe there is still room for improvement.

We hope that our research can provide some inspiring ideas for the development of this field.
